# Atypical Cases of Leptospirosis: Insights From Georgia

**DOI:** 10.1155/2024/1414417

**Published:** 2024-11-20

**Authors:** Arash Ghani Dehkordi, Salah Rabea Salah Al-Tamary, Ashraf Rabea Salah Al-Tamary, Hamza Rabea Salah Al-Tamary, Shams Samih Ahmad Albarari, Alaaldin Mohammad Zeyad Assad, Ghena Mohammad Othman Hamdan, Ia Mikadze

**Affiliations:** Department of Infectious Diseases, Tbilisi State Medical University, Tbilisi, Georgia

**Keywords:** atypical presentation, case report, diagnostic challenges, leptospirosis

## Abstract

Pathogenic *Leptospira* species are the source of leptospirosis, a common zoonotic infection that can cause a wide range of clinical manifestations, from minor flu-like symptoms to severe multiorgan failure. We present two peculiar cases of leptospirosis; they highlight the need for clinical awareness to improve patient outcomes and further knowledge of leptospirosis epidemiology and therapy by illuminating the difficulties in diagnosis and treatment. The first case involved a 30-year-old male presented with jaundice. Although he had no history of chronic illnesses, an exhaustive investigation was warranted due to his recent travel history and occupational contact. Laboratory tests revealed significantly increased levels of AST and ALT and positive *Leptospira* IgM serology. Remarkably, the patient refuted the traditional theory of leptospirosis transmission by denying direct animal interaction. After starting therapy with dexamethasone initially and adding doxycycline later, the patient's condition significantly improved; his jaundice resolved and his liver enzyme levels returned to normal. An outpatient follow-up after discharge was advised to assess liver and kidney function. The second case involved an 87-year-old woman with a fever, weakness and hypertension. Investigations revealed hepatosplenomegaly, raising the possibility of hypersplenism. She reported exposure to animals, particularly her dogs in her urban house. Surprisingly, her AST and ALT levels were normal. Lab tests also revealed thrombocytopoenia with normal APTT and prolonged PT. Serological tests indicated positive *Leptospira* IgM. Along with intravenous infusions, the patient's treatment plan comprised dexamethasone, enalapril and ceftriaxone to treat inflammation, hypertension and bacterial infection, respectively. Following a 20-day hospital stay, the patient's laboratory results and symptoms improved, leading to her discharge. Continuous follow-up recommended to monitor her recovery and prevent recurrence. These case studies emphasise the significance of taking leptospirosis into account when treating patients who do not have normal exposure histories yet present with unusual symptoms.

## 1. Introduction

All around the world, pathogenic leptospires cause leptospirosis, an acute bacterial septicaemic febrile illness that affects both humans and animals [1]. Leptospirosis is commonly referred to as a curable illness that is transmitted between animals and humans and is prevalent in economically disadvantaged nations located in temperate and tropical areas [2]. The majority of cases, specifically 73%, are concentrated in Southeast Asia, East sub-Saharan Africa, the Caribbean and Oceania [3]. *Leptospira* is a genus of spirochaetes that specifically belongs to the family Leptospiraceae. The organisms in this genus have a body diameter of 0.1 *μ*m and a length ranging from 6 to 20 *μ*m. *Leptospira* consists of several species, some of which are harmful. Leptospires are categorised into pathogenic (parasitic and infectious) and nonpathogenic strains (saprophytic and noninfectious) based on clinical criteria [4]. Infection occurs when individuals are directly or indirectly exposed to reservoir host animals that are infected with the disease. These animals carry pathogenic leptospires in their renal tubules and release them in their urine [5]. *Leptospira* infiltrates the body via lacerations or abrasions on the skin or through mucous membranes in the nose, eyes or throat. Phagocytes engulf spirochaetes, which can survive inside macrophages. The infected macrophages are shown to migrate away from the initial site of infection, indicating a potential alternate route for the spread of *Leptospira* [6]. The progression of leptospirosis is often categorised into two phases: the leptospiraemic phase, also known as the acute phase, and the immunological phase [3]. The diagnosis of *Leptospira* infection involves three distinct procedures: general laboratory results, indirect methods and direct methods [7]. The outcome of leptospirosis is contingent upon the seriousness of the condition, timely detection and immediate and intense medical intervention. Untreated, the patient may experience renal failure, meningitis, liver damage and respiratory difficulty. Death may occur in certain instances [8].

## 2. Case 1: Presentation

### 2.1. Initial Presentation and Patient History

A 30-year-old man presented to the infectious disease department with a one-week history of sudden onset of jaundice. He did not have any cardinal signs of inflammation such as fever and pain. The patient had no significant medical history or chronic illnesses. He had visited Germany and Poland six months earlier and then returned to his construction job, but he denied having any direct animal contact during this time. Over-the-counter drugs did not alleviate his symptoms.

### 2.2. Physical Examination (PE)

Upon PE, hepatomegaly and abdominal pain in the right hypochondrium with no cardinal signs of inflammation were revealed. Nevertheless, the patient's presentation did not specifically address certain classical signs and symptoms of leptospirosis, such as fever, myalgia and conjunctival suffusion. No particular gastrointestinal symptoms such as vomiting or diarrhoea which are common in patients with leptospirosis were found.

### 2.3. Laboratory Tests

The laboratory results are compiled in Tables 1–6.

All of the patients' full blood count (FBC) values—WBC, differential, RBC and platelet (PLT) counts—are generally within or very close to normal levels implying that there are no notable haematological anomalies that are usually connected to severe leptospirosis (see Table 1).

Alanine aminotransferase (ALT) and aspartate aminotransferase (AST): The patient's ALT and AST levels were substantially greater than usual, significantly surpassing the normal range (see Table 2). Increased ALT and AST values are often seen in leptospirosis because of the infection-induced hepatocellular damage [9].

Gamma-glutamyltransferase (GGT): The GGT level of the patient was greater than the upper limit of the normal range (see Table 2). When comparing leptospirosis to AST and ALT, elevated GGT levels are less common. While hepatic damage can cause GGT to rise, it is not as specific to leptospirosis as AST and ALT are [10].

Total bilirubin (TBIL) and direct bilirubin (DBIL): The patient had TBIL and DBIL levels that were much greater than usual (see Table 2). Because of cholestasis and hepatic injury, elevated bilirubin levels—especially DBIL (conjugated)—are frequently seen in leptospirosis [10].

Even though the patient's liver function tests (LFTs) revealed abnormalities that were consistent with hepatocellular injury, haemorrhagic manifestations—such as ecchymoses or petechiae—which can happen in severe cases of leptospirosis, were not observed [5].

The patient's LFTs revealed hepatic involvement; however, no specific reference was given to renal symptoms, which are frequent in acute kidney injury (AKI) associated with leptospirosis and include proteinuria and oliguria [1].

The patient's creatinine level was slightly below the typical range's lower boundary (see Table 3). AKI is a common complication of leptospirosis [11] and this impaired kidney function raises creatinine levels [12]. Given that the patient's creatinine level is below normal, this is atypical in the case of moderate to severe leptospirosis.

Similar to creatinine, urea levels can rise as a result of AKI in leptospirosis (see Table 3) [13]. However, a patient with urea level within the normal range is unusual for leptospirosis.

The patient's normal prothrombin time (PT) and activated partial thromboplastin time (APTT) indicated that there were no notable anomalies in either the extrinsic or intrinsic coagulation pathways (see Table 4).

The patient's absence of substantial coagulation abnormalities was further corroborated by the patient's normal international normalised ratio (INR) (see Table 4). Severe leptospirosis cases are usually associated with disseminated intravascular coagulation (DIC), which can result in extended PT and INR [14].

The patient had a fibrinogen (FIB) level that was slightly below normal (see Table 4). In severe circumstances, the consumption of FIB in DIC may cause a drop in FIB levels, which would extend the PT and INR. Nonetheless, the patient's normal INR and PT indicate that DIC and coagulopathy are unlikely [15].

The patient's alpha-fetoprotein (AFP) test result was within the normal range, ruling out hepatocellular carcinoma (see Table 5) [16].

The urine analysis revealed proteinuria with a protein level of 30 mg/dL, indicating renal involvement (see Table 6) [17].

The urine was noted to be cloudy with a specific gravity of 1.030 and a pH of 7.0, which were abnormal (see Table 6) [17].

The patient's clinical appearance and increased liver enzymes led to the first diagnosis of hepatitis. Although leptospirosis and hepatitis share certain clinical characteristics, the lack of conventional hepatitis risk factors and the resulting serological results for *Leptospira* IgM indicated a challenging presentation.

### 2.4. Ultrasound Imaging

The right lobe of the liver was measured to have a reticular oblique size of 180 mm (normal range < 150 mm). Similarly, the craniocaudal size of the left lobe of the liver was 107 mm (normal range < 100 mm).

### 2.5. Chest X-Ray

The lungs, heart and surrounding structures were all shown to be free of anomalies or pathological changes on the chest X-ray. There were no masses, consolidations or infiltrates seen on the radiograph.

### 2.6. Hepatitis Tests

A diagnostic problem arose from the patient's initial faintly positive anti-HCV test result. It did not verify continuous infection, even though it raised the possibility of past HCV exposure. The patient had negative results for hepatitis A, B and D, meaning that there were no recent or ongoing infections. Because the results were inconclusive, other diagnoses such as leptospirosis and autoimmune hepatitis were also considered.

### 2.7. Other Tests

The patient was not HIV-positive. Furthermore, ferritin levels were significantly greater than normal, ranging from 30 to 400 ng/mL, with a value of 1839.0 ng/mL. Increased ferritin levels could be a sign of liver illness, infection or inflammation, among other disorders. Ferritin levels may first remain unchanged or even increase as a result of the infection-induced inflammatory response. Ferritin levels may fall as the disease worsens. Haemolysis, inflammation and liver failure are a few possible influences on this decrease [15]. Additionally, the patient's total protein level of 68 g/L was within the normal range of 66–87 g/L, indicating that the blood protein levels were sufficient. Likewise, the patient's albumin (ALB) level of 44 g/L was within the normal range of 35–52 g/L, which suggested that protein production and liver function were both normal. This could suggest that although the patient sustained liver damage, it was not severe enough to have a major impact on the production of proteins.

### 2.8. Serological Test

The value of 24 in the first test result was greater than the permitted range of 15–20. The patient's diagnosis of leptospirosis was supported by increased IgM levels. Further confirmation of the existence of *Leptospira* IgM antibodies in the patient's serum—which indicates persistent or continuous infection—came from repeating the test and finding a higher score of 34.

Increased *Leptospira* IgM antibodies are a sign of a recent or ongoing illness. During the acute phase of leptospirosis, IgM antibodies usually increase and can be detected for a few weeks. Consequently, the patient's increased IgM levels are consistent with acute leptospirosis.

## 3. Differential Diagnosis

The patient initially presented with jaundice suggestive of hepatitis. His lab tests revealed markedly elevated liver enzymes (AST and ALT). Differential diagnoses encompassed acute viral hepatitis (A, B, C, D and E) and autoimmune hepatitis, but these were less likely due to the absence of autoimmune signs and negative viral hepatitis tests. Other infectious diseases, such as typhoid fever, brucellosis and Q fever, were considered given the patient's history and occupational exposure, but leptospirosis emerged as the leading diagnosis due to clinical context and positive *Leptospira* IgM serological test results. Noninfectious causes of liver damage, like autoimmune hepatitis and alcoholic liver disease, were ruled out based on clinical presentation and history. Leptospirosis was supported by the patient's occupation, recent travel and symptomatology, reinforcing its likelihood as the underlying condition.

## 4. Treatment

Tetracycline, laxatives, glucocorticoids, Hepa-Line therapy, Nacl, KCl and intravenous hydration were all part of the patient's treatment plan. Dexamethasone (4 mg/day), Hepa-Line therapy (20 mL/day), Duphalac (25 mg/day) and supportive care were the initial therapeutic components. Based on the serological findings, 100 mg of doxycycline twice daily (BID) was then started. During the patient's ten-day hospital stay, intravenous fluids such as Ringer's solution, NaCl and KCl were given. He showed clinical progress during his hospital stay, and he was released from the hospital in a stable state.

## 5. Outcome

Once the treatment plan was followed, the patient demonstrated significant clinical improvement, as evidenced by the elimination of jaundice, normalisation of liver enzymes and general improvement in symptoms. *Leptospira* IgM levels were shown to have decrease in subsequent laboratory tests, confirming the effectiveness of antibiotic therapy. Following these encouraging results, the patient was released from the hospital and given instructions for monitoring and follow-up at home.

## 6. Follow-Up

Outpatient visits were part of the follow-up strategy, which allowed for continued monitoring of renal and liver function as well as symptom alleviation. The patient was also cautioned to watch out for any repercussions, including the return of symptoms, reinfection or the development of long-term effects. The goal of this all-encompassing strategy was to guarantee the patient's ongoing health and to quickly take care of any changing medical requirements.

## 7. Case 2: Presentation

### 7.1. Initial Presentation and Patient History

An elderly woman, aged 87 years, presented with 38°C fever, light-headedness, weakness and high blood pressure for the past week. Medical history includes both chronic heart failure and arterial hypertension. She states that she takes the prescription drugs but provides no details about her medication schedule or adherence. The patient's exposure history included contact with animals, particularly dogs.

### 7.2. Clinical Findings

Upon PE, hepatosplenomegaly, which is mostly indicative of hypersplenism, was noticed.

## 8. Investigations

The investigations included haematological evaluation, including a FBC; bacteriological tests, including blood and urine cultures; LFTs; imaging studies, such as ultrasound; and serological tests.

There was no considerable leucocytosis, common in the early stages of leptospirosis (see Table 7). Nonetheless, the WBC count was mildly leukopenic, as it was at the lower end of the normal range. There were data from 201 patients with leptospirosis. Leucocyte counts and absolute neutrophil counts decreased during the initial 5 days of sickness (see Table 7), then increased until the conclusion of the second week. By the third day of fever, 75% of individuals had normal leucocyte counts. By the fifth day, leucocytosis was only present in 38.1% of cases, while leucopenia was rare [15].

Lymphocytes (LYMPHs): The lymphocyte count was below normal, with an overall decrease of 0.56 × 10^9^/L (see Table 7). Leptospirosis frequently causes lymphopenia, which may be a manifestation of immunological suppression or a reduction in LYMPHs as a result of systemic inflammation. In the same De Silva et al. trial, lymphopenia was detected in more than half of the patients on Day 5, but by Day 10, it had dropped to less than a quarter of the patients [15].

Monocytes (MONO): No appreciable deviations in monocyte levels are suggested by the number of MONO, which is within the normal range (see Table 7).

Basophils (BASO) and eosinophils (EO): Since both BASO and EO fall within the normal range, there is no evidence of either eosinophilia or basophilia (see Table 7).

Neutrophils (NEU): Neutrophilia is indicated by an increased neutrophil count (83.70%) (see Table 7). Neutrophilia, which represents the body's inflammatory reaction to infection, is frequently observed during episodes of severe leptospirosis and other acute bacterial infections. In patients with severe illness, total white cell and neutrophil counts were greater, while haemoglobin and haematocrit were significantly lower, according to the same De Silva et al. study [15].

Furthermore, the existence of microcytic, hypochromic red blood cells is suggested by the slightly greater MCV and increased RDW (see Table 7).

PLT count: Thrombocytopoenia is indicated by a markedly reduced PLT count (65 × 10^3^/*μ*L) (see Table 7). Leptospirosis is characterised by thrombocytopoenia, which is brought on by immune-mediated PLT destruction, bone marrow suppression and endothelial activation. Thrombocytopoenia, with PLT count below 150 × 10^9^/L, was observed in 56.76% of patients on the third day, increasing to 73.8% on the fifth day. Thrombocytopoenia was observed in 80.7% of cases at some point of the disease [15].

C-reactive protein (CRP): At 38 mg/L, the CRP level is noticeably higher than the usual range (< 5 mg/L) (see Table 7). A strong inflammatory response, which is common in leptospirosis, is indicated by elevated CRP levels. Acute-phase reactant CRP rises in reaction to tissue damage, infection or inflammation. CRP rise in leptospirosis is indicative of the bacterial infection–induced systemic inflammatory response [18].

TBIL: The patient had a TBIL level that was greater than normal (≤ 24.0 *μ*mol/L), at 42.50 *μ*mol/L (see Table 8). Leptospirosis is frequently associated with elevated bilirubin levels, which may be caused by cholestasis or hepatic dysfunction brought on by inflammation or hepatocellular damage [5].

The levels of AST and ALT are both within the normal range; AST is 20 U/L (normal ≤ 32 U/L) and ALT is 11.30 U/L (normal ≤ 35.0 U/L) (see Table 8). In leptospirosis, normal ALT and AST levels indicate minimal hepatocellular damage, which is rather uncommon because increased transaminases are often seen as a result of hepatic inflammation [3].

GGT: At a level of 26 U/L (normal range: 5–36 U/L), the GGT test does not significantly show signs of hepatobiliary dysfunction or biliary blockage (see Table 8). An absence of cholestatic characteristics, which are frequently observed in severe types of leptospirosis, is consistent with normal GGT levels. Damage to hepatocytes and cholangiocytes is indicated if both alkaline phosphatase (ALP) and GGT are increased and all other potential causes of liver injury (such as alcoholism and infections) are ruled out. Damage to the bile canaliculus and cholangiocytes is indicated if GGT is increased while ALP is not. When ALP is raised but GGT is not, liver injuries are frequently ruled out [19].

Other liver enzymes: The LFT results do not provide further information on liver enzymes such as lactate dehydrogenase (LDH) and ALP.

The result of the APTT, which is 29.7 s, is within the usual range (25.4–43.0 s) (see Table 9). Normal APTT indicates the lack of intrinsic pathway coagulation factor deficits, which are not usually significant aspects of leptospirosis but can be affected in some cases [20].

The INR was at 2.60 (normal range: 0.90–1.30) and the PT was prolonged by 30.5 s (normal range: 12.1–16.5 s) (see Table 9). An extended PT and increased INR are indicative of compromised extrinsic pathway coagulation, usually resulting from deficiencies in factors dependent on vitamin K (Factors II, VII, IX and X).

FIB: Hypofibrinogenemia is indicated by a reduced FIB level of 1.9 g/L (normal range: 2.0–4.0 g/L) (see Table 9). Diminished FIB concentrations align with consumptive coagulopathy observed in severe leptospirosis cases, especially those exacerbated by DIC. Nevertheless, more assessment is required to verify the existence of DIC [15].

Proteinuria was present because the urine analysis's 100 mg/dL protein level was high (see Table 10). When leptospirosis is severe or when there is renal involvement, proteinuria may especially arise.

Turbidity is described as cloudy, which may indicate the presence of particulate matter such as bacteria, crystals or pieces of cell debris (see Table 10). A urinary tract infection or inflammation may be indicated by cloudy urine, which is an indeterminate finding.

### 8.1. Ultrasound

Ultrasonography revealed 180 × 76 mm for the spleen (normal: 130 × 40 mm), confirming the existence of hepatosplenomegaly. Severe leptospirosis frequently manifests as hepatosplenomegaly, which is usually explained by hepatic and splenic congestion, inflammation and oedema caused by systemic effects of the infection [21].

### 8.2. Chest X-Ray

The results from the chest X-ray showed no pathology, indicating that there were no notable abnormalities in the lung fields, mediastinal structures or pulmonary parenchyma. In severe cases of leptospirosis, pulmonary involvement, including interstitial infiltrates, pulmonary haemorrhage or acute respiratory distress syndrome (ARDS), can develop. Although there is a constellation of different manifestations in this patient, each indicating a different intensity of the disease, the absence of acute abnormalities on chest X-ray is uncommon in severe leptospirosis patients [22].

### 8.3. Blood and Urine Biochemical Tests

#### 8.3.1. Blood Culture

Blood culture reveals no growth of any bacterial pathogens.

#### 8.3.2. Urine Culture

The results of the urine culture also showed sterility, with no bacterial pathogen development noted. Like blood cultures, intermittent shedding of bacteria in urine and the limitations of traditional culture methods can make it challenging to achieve a positive urine culture for *Leptospira*. Even if the urine culture results are negative, leptospirosis should still be considered a possible diagnosis because further clinical and laboratory results support this diagnosis.

### 8.4. Other Tests

The patient did not appear to have any concomitant viral infections, as indicated by the negative results of the serological tests for HIV and hepatitis viruses, including HCV, HAV and HBV. These results help rule out other possible explanations for the patient's clinical presentation and reinforce the focus on leptospirosis as the most likely diagnosis.

### 8.5. Serological Tests

The absence of antibodies against *Leishmania*, *Brucella* and *Borrelia* is indicated by negative serological test findings. Nonetheless, the patient's diagnosis of leptospirosis is strongly supported by the positive *Leptospira* IgM antibody test result. *Leptospira* IgM antibodies were detected in the patient's blood sample by the *Leptospira* serological test, with a titre of 80 (normal range: 15–20). This result suggested acute or recent *Leptospira* infection. Strong evidence confirming the diagnosis of leptospirosis in the patient was provided by elevated IgM antibody titres against *Leptospira*.

## 9. Differential Diagnosis

Fever and multisystem involvement in a patient can be caused by a number of infectious and noninfectious disorders. These include bacterial infections like brucellosis and typhoid fever, as well as viral infections such as hepatitis and dengue fever. Possible causes include malignancies, autoimmune disorders and abnormalities in metabolism. Hepatosplenomegaly and elevated liver enzymes indicate hepatic involvement, which raises the possibility of diseases such acute viral hepatitis or drug-induced liver injury. Anaemia and thrombocytopoenia may point to underlying haematological disorders; however, given the patient's medical history, it is important to rule out the possibility of worsening of cardiovascular problems. On the other hand, leptospirosis becomes the main worry when exposed to potentially contaminated settings and a positive *Leptospira* IgM antibody test, which is consistent with the reported clinical manifestations.

### 9.1. Treatment

During her 20-day hospital stay, the patient received rigorous medical care, including drug delivery, fluid infusions and close monitoring of her vital signs and test results. The goal of the treatment plan was to promote the patient's overall health and recovery while also treating the underlying infection and controlling any related consequences.

The patient's first treatment plan included the following elements. Dexamethasone 4 mg twice a day: As directed by the haematologist, this medication is taken to control the immune system and minimise inflammation. Enalapril 10 mg once daily: used to treat hypertension and chronic heart failure by widening blood arteries and enhancing heart function. Ceftriaxone 1 g twice a day: an antibiotic used to treat the underlying *Leptospira*-caused bacterial illness. Intravenous infusions: To maintain fluid and electrolyte balance, treat dehydration and avoid problems, normal saline (NaCl), potassium chloride (KCl) and Ringer's solution were administered. To target the *Leptospira* bacteria, 100 mg of doxycycline twice daily was administered when leptospirosis was confirmed.

### 9.2. Clinical Outcomes

The patient had satisfactory clinical outcomes after an extensive treatment plan. There was a noticeable improvement in symptoms. Laboratory tests improved enabling her to be discharged.

### 9.3. Follow-Up

The patient needed continuous follow-up treatment after being released from the hospital to avoid experiencing recurrence of leptospirosis.

## 10. Discussion

Our experience with these individuals highlights how leptospirosis is a constantly changing disease and highlights the significance of keeping a high index of suspicion, especially in areas where the illness is endemic. Healthcare professionals can improve patient outcomes and avoid harmful side effects by identifying and treating leptospirosis' variety of clinical manifestations as soon as possible.

*Leptospira* infiltrates the host and easily enters the bloodstream, causing bacteraemia. Bacteria disseminate to organs and tissues via the bloodstream, establishing colonies in the spleen, liver, lungs and kidneys, where they proliferate and propagate. The peak concentration of *Leptospira* in the bloodstream can be identified on the fifth day following infection. The incubation period for leptospirosis typically ranges from 5 to 14 days; however, it can occasionally extend up to 30 days [23]. Leptospirosis can manifest in two well-defined clinical syndromes, namely, icteric or anicteric. Anicteric syndrome is a condition that resolves on its own and manifests as a nonspecific flu-like disease. The onset typically occurs abruptly and may manifest as a headache, cough, nonpruritic rash, fever, rigours, myalgia, anorexia and diarrhoea. The duration of this disease may span a few days until the fever subsides. This variant of the disease is rarely fatal and accounts for nearly 90% of all recorded instances of leptospirosis. The anicteric syndrome may exhibit a recurrence, known as the immunological stage, which can occur many days later and may be accompanied by aseptic meningitis. These individuals have the potential to achieve complete recovery, but they may experience persistent, episodic headaches. Weil's disease is the conventional term for the icteric phase of leptospirosis. This is a profound illness characterised by symptoms such as fever, AKI, jaundice, haemorrhage and respiratory distress. The icteric phase can also affect the heart, central nervous system (CNS) and muscles. This sickness typically manifests as a severe condition and can last for several weeks or even months in the event of the patient's survival [24]. Fever is a positive yet nonspecific reaction of the body to infections and other pyrogens, resulting in the production of cytokines by immune cells, which in turn leads to an increase in body temperature mediated by brain proteins. Cytokines may be broadly categorised into two groups: proinflammatory cytokines and anti-inflammatory cytokines. IL-1, TNF-*α* and IL-6 are cytokines that promote inflammation [25]. When there are elevated levels of leptospiraemia during an infection, the body's innate immune system ultimately triggers reactions in both the affected tissues and throughout the body. These responses may result in severe outcomes, such as a state similar to sepsis or multiorgan failure. Those suffering from severe leptospirosis exhibit indications of a ‘cytokine storm' characterised by elevated levels of IL-6, TNF-alpha and many other cytokines compared to those with milder symptoms [5]. Gastrointestinal symptoms, which may encompass abdominal pain, nausea, vomiting and diarrhoea, can lead to dehydration in individuals with high-output nonoliguric renal failure resulting from leptospirosis [5]. Conjunctival suffusion is a distinctive observation that often occurs during the third and fourth days [3]. Conjunctival suffusion is due to an increase in blood circulation. In leptospirosis, the normally invisible blood veins of the conjunctiva become permeable, allowing red blood cells to penetrate the conjunctival tissue. Vessel dilation occurs as a result of the immunological response, leading to an increase in blood flow. The sclera has a noticeable red hue and enhanced visibility of blood vessels [26]. Leptospirosis can be considered a kind of haemorrhagic septicaemia. As a result, the primary observations related to blood vessels, particularly those in the microcirculatory system, are crucial for understanding their development. VE-cadherin is a crucial element of endothelial adherence junctions responsible for maintaining the integrity of blood vessels. Pathogenic leptospires can attach themselves to VE-cadherin present in human mammalian cell cultures as well as the endothelial cells that line these blood vessels. The attachment of pathogenic leptospires to host cells via cadherins does not necessarily result in intracellular invasion. Experimental and autopsy data indicate that there is a change in the permeability of the cell membrane. This change was observed using CD34 and VE-cadherin immunohistochemical detection in the pulmonary circulation of autopsied patients who experienced significant pulmonary haemorrhage. The observed morphological changes, especially in the case of VE-cadherin, can explain the increased ability of blood vessels to allow substances to pass through in conditions that affect the organisation of adherens junctions [1]. Leptospirosis causes the disarray of hepatocytes by disrupting the intercellular junctions. This leads to a moderate increase in hepatic transaminases and the obstruction of DBIL, resulting in jaundice [23]. Leptospirosis often affects the kidney, with tubulointerstitial nephritis being the most prevalent clinical and pathological presentation. Leptospirosis commonly leads to nonoliguric AKI in humans, accompanied by hypokalaemia and the loss of sodium and magnesium. Leptospirosis can lead to complications such as leptomeningitis and myocarditis. In leptospirosis, the heart commonly exhibits vascular damage, which is typically defined by segmental lesions on the walls of the coronary artery and its branches. The primary and most significant abnormality in leptospirosis is vascular injury, specifically affecting the endothelial cell as a whole. Aortitis is an intriguing vascular abnormality observed in leptospirosis [1]. The majority of bleeding symptoms are rather mild, such as petechiae, ecchymoses and epistaxis. Nevertheless, certain patients have severe gastrointestinal bleeding (melaena or haematemesis) or pulmonary haemorrhage. Thrombocytopoenia commonly occurs, albeit typically not severe enough to result in spontaneous bleeding. Research conducted in the Netherlands on severe leptospirosis revealed that all patients exhibited coagulation abnormalities, namely, an increase in PT. Furthermore, it was shown that the extent of PT prolongation was directly linked to the severity of bleeding symptoms [5]. Neuroleptospirosis mainly presents as aseptic meningitis, with additional neurological symptoms including encephalitis, intracranial haemorrhage, cerebellitis and myelitis. Leptospirosis is an infrequent cause of aseptic meningitis, accounting for only 5%–13% of cases [27].

In conclusion, by presenting two cases of leptospirosis, this report has shed light on how the disease's clinical and epidemiological landscape is changing. Our results highlight a number of significant features of leptospirosis in the contemporary period. First, leptospirosis has become more prevalent and may extend farther into metropolitan areas, as evidenced by its geographical spread. Second, our examples show that leptospirosis affects people of all ages and genders, emphasising the significance of taking this diagnosis into account in a patient community that is heterogeneous. Moreover, leptospirosis can cause a variety of clinical signs and symptoms that affect different organ systems. While some cases could show up with standard symptoms like fever and jaundice, others might show up with unusual characteristics or even just isolated symptoms like a persistent fever without any systemic involvement. Serological testing for leptospirosis must be a part of the diagnostic process for suspected cases due to the disease's variable presentation and ability to mimic other illnesses. This strategy can help with prompt diagnosis and proper treatment initiation, which reduces the risk of problems. It should be used in conjunction with comprehensive evaluation of the epidemiological history.

## Figures and Tables

**Table 1:** Full blood count.

Test	Result	Unit	Normal range
WBC	7.00	10^9^/L	4.00–11.00

LYMPH	28.1	%	20.0–45.0
	1.97	10^9^/L	1.00–5.00

MONO	2.8	%	2.0–12.0
	0.20	10^9^/L	0.10–1.00

ALY/LUC	1.3	%	≤ 1.5
	0.09	10^9^/L	≤ 0.20

EO	1.70	%	1.00–5.00
	0.12	10^9^/L	0.10–0.25

BASO	0.90	%	≤ 1.00
	0.06	10^9^/L	≤ 0.10

NEU	66.50	%	50.00–70.00
	4.65	10^9^/L	2.00–7.00

LIC/LI	0.90	%	0.01–1.50
	0.06	10^9^/L	0.01–0.20

RBC	5.46	10^12^/L	4.50–5.80

HGB	15.4	g/dL	13.5–17.0

HCT	45.3	L/L	40.0–50.0

MCV	82.9	fL	80.0–98.0

MCH	28.20	pg	26.00–34.00

MCHC	34.00	g/dL	31.00–35.00

RDW	15.30	%	10.00–16.00

PLT	168	10^3^ *μ*L	150–380

MPV	10.70	fL	7.4–10.4

PCT	0.179	%	0.200–0.500

PDW	22.80	%	10.00–18.00

CPR	4	Mg/L	< 5

ESR	Not measured	Mm/h	≤ 27

**Table 2:** Liver function test.

Test	Result	Unit	Normal range
GOT (AST)	1293	U/L	≤ 40
GGT (*γ*-GT)	316	U/L	8–61
TBIL	271.10	umol/L	≤ 24.0
GPT (ALT)	1783.00	U/L	≤ 50.0
DBIL	137.00	umol/L	≤ 5.0
LIPASE	24	U/L	13–60

**Table 3:** Renal function test.

Test	Result	Unit	Normal range
CREA	52.50	*μ*mol/L	54.00–104.00
Urea	4.50	mmol/L	2.76–8.07
Ammonia (NH_3_)	41.50	*μ*mol/L	16.00–60.00
LDH	421	U/L	135–225

**Table 4:** Coagulation panel.

Test	Result	Unit	Normal range
APTT	30.2	sec	25.4–43.0
PT	13.4	sec	12.1–16.5
INR	1.16	sec	0.90–1.30
PI	81.00	%	76.00–104.00
FIB	1.9	g/L	2.0–4.0 ↓
TT	Not measured	sec	15.00–22.00

**Table 5:** Alpha-fetoprotein.

Test	Result	Unit	Normal range
AFP	4.55	U/mL	< 5.80

**Table 6:** Urine analysis.

Test	Result	Normal range
Tot. Amount	60 mL	
Colour	Brown	Yellow
Turbidity	Cloudy	Clear
Specific gravity	1030	1.015–1.025
pH	7.0	5.0–6.5
Leucocytes	Negative	Negative
Nitrates	Negative	Negative
Protein	30 mg/dL	Negative
Glucose	100 mg/dL	Negative
Ketones	Trace	Negative
Urobilinogen	1.0 E.U./dL	0.2–1.0 E.U./dL
Bilirubin	Large	Negative
Blood	Trace-intact	Negative

**Table 7:** Full blood count.

Test	Result	Unit	Normal range
WBC	4.19	10^9^/L	4.00–11.00

LYMPH	13.3	%	20.0–45.0
	0.56	10^9^/L	1.00–5.00

MONO	2.3	%	2.0–12.0
	0.10	10^9^/L	0.10–1.00

ALY/LUC	0.3	%	≤ 1.5
	0.01	10^9^/L	≤ 0.20

EO	0.40	%	1.00–5.00
	0.02	10^9^/L	0.10–0.25

BASO	0.30	%	≤ 1.00
	0.01	10^9^/L	≤ 0.10

NEU	83.70	%	50.00–70.00
	3.50	10^9^/L	2.00–7.00

LIC/LI	0.10	%	0.01–1.50
	0.01	10^9^/L	0.01–0.20

RBC	3.49	10^12^/L	4.50–5.80
HGB	11.5	g/dL	13.5–17.0

HCT	33.2	L/L	40.0–50.0

MCV	95.0	fL	80.0–98.0

MCH	32.90	pg	26.00–34.00

MCHC	34.60	g/dL	31.00–35.00

RDW	16.40	%	10.00–16.00

PLT	65	10³ *μ*L	150–380

MPV	9.60	fL	7.4–10.4

PCT	0.062	%	0.200–0.500

PDW	15.50	%	10.00–18.00

CPR	38	Mg/L	< 5

ESR	Not measured	Mm/h	≤ 27

**Table 8:** Liver function test and partial renal test (creatinine and urea).

Test	Result	Unit	Normal range
CREA	66.20	*μ*mol/L	45.00–84.00
Urea	6.62	mmol/L	2.76–8.07
GOT (AST)	20	U/L	≤ 32
GGT (*γ*-GT)	26	U/L	5–36
TBIL	42.50	umol/L	≤ 24.0
GPT (ALT)	11.30	U/L	≤ 35.0

**Table 9:** Coagulation tests.

Test	Result	Unit	Normal range
APTT	29.7	sec	25.4–43.0
PT	30.5	sec	12.1–16.5
INR	2.60	sec	0.90–1.30
PI	30.00	%	76.00–104.00
FIB	1.9	g/L	2.0–4.0
TT	Not measured	Sec	15.00–22.00

**Table 10:** Urine analysis.

Test	Result	Normal range
Tot. Amount	40 mL	
Colour	Yellow	Yellow
Turbidity	Cloudy	Clear
Specific gravity	1020	1.015–1.025
pH	6.5	5.0–6.5
Leucocytes	Negative	Negative
Nitrates	Negative	Negative
Protein	100 mg/dL	Negative
Glucose	Negative	Negative
Ketones	Negative	Negative
Urobilinogen	1.0 E.U./dL	0.2–1.0 E.U./dL
Bilirubin	Negative	Negative
Blood	Large	Negative

## Data Availability

The data that support the findings of this study are available from the corresponding author upon reasonable request.

## Nomenclature

WBC: White blood cells
LYMPHs: Lymphocytes
MONO: Monocytes
ALY/LUC: Atypical lymphocytes/large unstained cells
EO: Eosinophils
BASO: Basophils
NEU: Neutrophils
LIC/LI: Lymphocytes in cerebrospinal fluid/left index
RBC: Red blood cells
HGB: Haemoglobin
HCT: Haematocrit
MCV: Mean corpuscular volume
MCH: Mean corpuscular haemoglobin
MCHC: Mean corpuscular haemoglobin concentration
RDW: Red cell distribution width
PLTs: Platelets
MPV: Mean platelet volume
PCT: Plateletcrit
PDW: Platelet distribution width
CPR: C-reactive protein
ESR: Erythrocyte sedimentation rate
GOT (AST): Glutamic-oxaloacetic transaminase (aspartate aminotransferase)
GGT (Γ-GT): Gamma-glutamyltransferase
TBIL: Total bilirubin
GPT (ALT): Glutamate pyruvate transaminase (alanine aminotransferase)
DBIL: Direct bilirubin
CREA: Creatinine
LDH: Lactate dehydrogenase
APTT: Activated partial thromboplastin time
PT: Prothrombin time
INR: International normalised ratio
PI: Platelet index
FIB: Fibrinogen
TT: Thrombin time
AFP: Alpha-fetoprotein
